# Clinical and Microbiological Evaluation of the Chemomechanical Caries Removal Agents in Primary Molars

**DOI:** 10.7759/cureus.31422

**Published:** 2022-11-12

**Authors:** Kartik Choudhary, Abhishek Gouraha, Mayank Sharma, Pratibha Sharma, Manoj Tiwari, Ajay Chouksey

**Affiliations:** 1 Department of Pedodontics and Preventive Dentistry, Rishiraj College of Dental Sciences and Research Centre, Bhopal, IND; 2 Department of Oral Pathology and Microbiology, Triveni Institute of Dental Sciences, Hospital and Research Centre, Bilaspur, IND; 3 Department of Pediatric and Preventive Dentistry, Mansarovar Dental College, Bhopal, IND; 4 Department of Orthodontics and Dentofacial Orthopedics, Mansarovar Dental College, Bhopal, IND; 5 Department of Periodontics, Mansarovar Dental College, Bhopal, IND

**Keywords:** carisolv, primary molars, microbial evaluation, clinical efficacy, chemomechanical caries removal agents

## Abstract

Background: Chemomechanical caries removal (CMCR) is a noninvasive procedure that uses a chemical substance to remove the diseased dentin. The natural dental architecture is also preserved using this technique, preventing patient discomfort and pulp irritation. This method of eliminating caries is based on disintegration. This method removes soft carious dentin using chemical agents and non-traumatic mechanical force. This study was carried out to evaluate the clinical and microbiological evaluation of the chemomechanical caries removal agents in primary molars.

Methods: For the elimination of caries, teeth in category I (polymer bur category) were treated with Smartburs II® (SS White Dental, Lakewood, New Jersey, United States) (n = 40). Teeth treated with the new Carisolv® technology (Mediteam, Sweden) to remove caries (n = 40). Teeth were treated for removal cavities in category III (conventional group) using excavators and carbon steel low-speed round burs from Dentsply Maillefer in Switzerland (n = 40). There was an evaluation of the efficacy of caries removal, the time required for caries removal, patient satisfaction, and microbial culture assessment.

Results: In comparison to the other two categories, the conventional category median caries detector dye values were considerably lower (p value<0.05). There was no substantial difference observed between study participants belonging to category I and category II with a p-value greater than 0.05. Time taken during the removal of caries was substantially greater in category I (455.46±73.7) as compared to category II (129.21±44.18) and the traditional category (113.26±37.7). The value of significance was less than 0.05. Contrarily, no discernible difference was observed between category I and category III (the p-value was greater than 0.05). In comparison to the other two categories, the conventional group's median facial expression scale scores were substantially higher (p = 0.02). In comparison, there was no discernible difference between categories I and II (p = 0.65). It was observed that there was no substantial variation in three categories regarding the number of colonies of bacteria prior to the eradication of caries (p-value greater than 0.05). After caries had been removed, the number of living bacterial colonies in category I was noticeably greater than those in the other two categories. However, there was no discernible variation between category II and category 1 (p-value greater than 0.05).

Conclusion: The mechanical approach has the highest clinical efficacy for removing caries. Carisolv required the most time to remove cavities. Patient satisfaction levels were greater with Carisolv than with the mechanical approach. It was also observed that Carisolv as well as the mechanical technique had greater antibacterial effectiveness.

## Introduction

In pediatric patients, caries involving primary molars is a significant issue. It is a complex illness brought on by the interaction of host, environmental, and behavioral variables. It results in the decalcification of the tooth's inorganic components and the destruction of its organic components [1].

There are two successive layers of caries involving dentin. These layers are distinct from one another in terms of their clinical characteristics, chemical compositions, and structural features at the microscopic level. The layer present at the outer side is considered infected dentin. It exhibits considerable bacterial invasion, permanent collagen fiber breakdown, and alteration of the architecture of dentinal tubules. The layer present on the inner side is considered affected dentin. It exhibits decalcification involving dentin found between dentinal tubules, deposition of crystals inside dentinal tubules, lighter degradation of the collagen matrix, and a reduced level of bacterial colonies despite the potential discoloration. The innermost layer is also more resilient to proteolytic degradation and the advancement of caries. As a result, the impacted dentin should be maintained while the infected dentin portion should be removed, albeit it is still difficult to distinguish between the two and only eliminate the infected dentin [2].

In pediatric dentistry, employing rotational devices to treat tooth cavities is troublesome. There are many drawbacks, including the patients' sense of pain, the need for local anesthetic, and the needless impairment of the dental tissues caused by the elimination of both infected dentin as well as affected dentin. Additionally, it may cause pulp exposure and have harmful heat impacts on pulpal tissue [3].

In the past few decades, innovations in operative dentistry and dental materials have changed the way that caries are treated in the field of dentistry. The current standard is to use adhesive restoration and minimum cavity preparation methods to keep cavities as tiny as possible. So, maintaining the structure of natural teeth as much as possible is the objective [4].

Chemomechanical caries removal (CMCR) is a noninvasive procedure that uses a chemical substance to remove the diseased dentin. The natural dental architecture is also preserved using this technique, preventing patient discomfort and pulp irritation. This method of eliminating caries is based on disintegration. This method removes soft carious dentin using chemical agents and non-traumatic mechanical force [5]. Polymer burs are another noninvasive cavity removal technique. The manufacturer claims that the polymer bur, a specific rotary tool composed of a specially formulated polymer substance, precisely eliminates infected dentin without slicing the normal dentin. It possesses a hardness that was intentionally developed to be greater than infected dentin's but lower than affected dentin's hardness. As a result, the bur can preserve the tougher, healthier dentin while removing the soft, carious dentin preferentially [6].

This study was carried out in order to examine and evaluate the clinical and microbiological effects of the chemomechanical caries removal agents in primary molars.

## Materials and methods

The study's design

A total of 120 compliant, healthy kids were included in this randomized, single-blinded clinical trial from the pediatric dental clinic. One tooth (primary molar) only was examined in each subject. Ethical approval was taken from a committee of Rishiraj College of Dental Sciences and Research Centre, Bhopal, India, with IRB number EC/NEW/INSI/2020/1299 (National Ethics Committee Registry for Biomedical and Health Research [NECRBHR]).

Inclusion requirements include pediatric patients between the ages of four and eight who are in good health and cooperative and who have Class I (occlusal) chronic caries affecting the upper portion of the dentin in the deciduous molars, as reflected by preliminary radiographs. There was no evidence of symptoms reflecting excruciating inflammation of the pulp or symptoms indicative of necrosis of the pulp.

Exclusion standards were for children who are disobedient, kids with poor health, kids that require specialized care, proximal carious teeth, flaws in tooth development, and teeth with prior restorations.

A written agreement signed by the parents and the kid was used to obtain their consent to participate in the study. Following the envelope randomization procedure, pediatric patients who fulfilled the standards of inclusion were randomly divided into three categories (40 study participants in each category and 120 total participants). The youngster was given the opportunity to choose at random from one of the study's caries eradication techniques once the parents had given their permission.

For the elimination of caries, teeth in category I (polymer bur category) were treated with Smartburs II® (SS White Dental, Lakewood, New Jersey, United States) (n = 40). Teeth were treated with the new Carisolv® technology (Mediteam, Sweden) to remove caries in category II (n = 40). Teeth were treated for removal of cavities in category III (conventional group) using excavators and carbon steel low-speed round burs from Dentsply Maillefer in Switzerland (n = 40).

Procedures

The following steps were taken with the kids. It was determined by the preliminary radiograph that caries affected the top half portion of the dentin.

Patient Preparedness and Management 

Strong rapport and nonverbal cues helped develop communication with the kids. For patients in categories I and II, a topical anesthetic cream (20% benzocaine) was all that was required to make applying the rubber dam painless. Patients assigned to category III received local anesthesia after two minutes of benzocaine 20% topical anesthetic gel applied to the dry mucosa.

An elastic dam was used to isolate every tooth. A high-speed drill was used to create the cavity's outline form in order to improve accessibility and visibility as well as to eliminate the unsupported enamel edges. Using a sterilized spoon excavator, a preliminary reference specimen of the dentin affected by caries was removed from the lesion. A sterile vial filled with distilled water was immediately used to transfer the dentin sample for microbial culture. Then, caries were eliminated in accordance with the group's type.

Category I (Polymer Bur Group)

According to the extent of the carious lesion, decaying dentin of sizes RA4, RA6, and RA8 was removed with SS White Smartburs II® employing a low-speed handpiece at five thousand revolutions per minute (RPM) to ten thousand RPM. Following the suggestions provided by the manufacturer, caries was dug using circular, gentle brush strokes from the center and top of the lesion to its edge, stopping once the equipment edge got blunted, observed macroscopically, and it was not capable of removing the carious dental tissue, confirmed with an explorer. Any remaining decay was removed with a new Smartburs II® if necessary.

Category II (Carisolv Group)

In accordance with the suggestions provided by the manufacturer, the gel was extensively soaked into the carious dentin for a duration of thirty seconds after being administered directly to the cavity. The gel was then washed with water once the softer portion of dentin was removed, employing manual excavators for removing teeth. The tactile approach was used to continue these steps until no soft portion of dentin was found.

Category III (Conventional Group)

On the basis of the architecture of the carious lesion affecting dental tissues, the dentin was eliminated by employing caries excavators as well as round carbon burs. The size of the burst involved was ISO type 012, ISO type 014, and ISO type 016. A handpiece was used with water coolant. When the explorer could no longer feel the soft dentin, the caries eradication was finished. Using only a sterile spoon excavator, a specimen of a carious portion of dentin was extracted from the deepest portion of the floor of the cavity in each group and deposited in a sterile container filled with distilled water. Dentin particles in an adequate quantity were acquired for bacterial consortium and assessment. Caries detector dye (Ultradent, South Jordan, Utah, United States) was applied for 10 seconds to identify residual caries, and then the area was rinsed with water for a duration of ten seconds. All teeth that went through treatment with different approaches in the study were rebuilt using resin-modified glass ionomer (RMGI) (GC Fuji, Tokyo, Japan), and patients were followed up for a week to assess any complaints they may have had.

Evaluation

Caries detector dye was utilized for a duration of 10 seconds to identify residual caries, and then the area was cleaned with water for 10 seconds. The recommendations outlined by Munshi et al. were utilized [7]. Effectiveness is rated as either completely effective or incomplete effectiveness and given a numerical score of 0, 1, 2, 3, or 5.

Time

Utilizing a stopwatch, the amount of time taken during each treatment was measured in seconds beginning with the elimination of caries.

Patient Satisfaction

Utilizing the Facial Image Scale, it was assessed. After each technique, the kids were instructed to indicate which face they identified with the most. Applying a score of 1 to the happiest face and 5 to the unhappiest face allowed for scoring.

Microbiological Evaluation

The acquired dentin specimens were promptly sent to the lab for bacterial culture and assessment in highly sterilized Eppendorf tubes containing 1 mL of normal saline. A liquid Luria-Bertani (LB) culture was used to dilute the collected samples before they were incubated on LB plates of agar. At 37°C, the LB plates of agar underwent a two-day aerobic incubation period. Following that, the total count of bacterial colonies per specimen was tabulated. It was represented in the form of CFU (colony forming units) as the number of colonies multiplied by (1/dilution).

Statistical Analysis 

The data was collected in the form of a mean with standard deviations. Intergroup differences were evaluated using the Kruskal-Wallis H test (non-parametric analysis of variance [ANOVA]). Intragroup differences were compared using the Mann-Whitney U test. The confidence level was maintained at 95% with the result that a P-value below 0.05 indicated a statistically significant association. All statistical analyses were carried out using IBM SPSS Statistics for Windows, Version 22.0 (Released 2013; IBM Corp., Armonk, New York, United States).

## Results

Results of clinical evaluation

Table 1 shows the sample distribution by the children's ages and genders.

**Table 1:** Sample distribution based on the kids' ages and genders SD- standard deviation

Category	Age (mean ± SD)	Gender	
		Male No (%)	Female No (%)
Category I	5.67 ± 1.43	16 (40)	24 (60)
Category II	6.61 ± 1.51	20 (50)	20 (50)
Category III	5.66 ± 1.58	18 (45)	22 (55)

Results of Efficacy (Caries Identification Dye Scores)

Comparing the results among three study groups for the scores of caries detection by caries identification dye is shown in Table 2. In comparison to the other two categories, the conventional category median caries detector dye values were considerably lower (p-value < 0.05). There was no substantial difference observed between study participants belonging to category I and category II with a p-value greater than 0.05.

**Table 2:** Caries detector dye results for the groups under study *: statistically significant

Category	Median (Min–Max)	Mann-Whitney (MW) test
Category I	1.5 (1–3)	P1 = 0.737
Category II	1.5 (0–4)	P2 = < 0.001
Category III	1.0 (0–2)	P3 = 0.001*
P value	<0.002*	

Results of Time

Time taken during removal of caries was substantially greater in category I (455.46 ±73.7) as contrasted to category II (129.21± 44.18) and the traditional category (113.26 ± 37.7). The value of significance was less than 0.05. Contrarily, no discernible difference was observed between category I and category III (the p-value was greater than 0.05).

Results of Patient Satisfaction (Facial Image Scale)

A comparison of the data of the three categories regarding facial expression scale gradings is shown in Table 3. In comparison to the other two categories, the conventional group's median facial expression scale scores were substantially higher (p =0.02). In contrast, there was no discernible difference observed between category I and category II (p=0.65).

**Table 3:** Scores on the facial image scale for the groups under study *: statistically significant

Category	Median (Min–Max)	Mann-Whitney (MW) test
Category I	1.5 (1–4)	P1 = 0.791
Category II	1.5 (1–4)	P2 = 0.038*
Category III	2.5 (1–6)	P3 = 0.02*
P value	0.026*	

Results of Microbiological Assessment

A comparison between the data of the number of living bacteria prior to the eradication of caries by the three approaches and the data of the number of living bacteria after is shown in Table 4. Additionally, it compares the total number of live bacteria after caries extraction using the three approaches. It was observed that there was no substantial variation in three categories regarding the number of colonies of bacteria prior to the eradication of caries (p-value greater than 0.05). After caries had been removed, the number of living bacterial colonies in category I was noticeably greater than those of the other two categories. However, there was no discernible variation between category II and category 1 (p-value greater than 0.05).

**Table 4:** The three groups' average values for the total viable bacterial count before and after caries removal *: statistically significant

Category	Before	After	
Category I	18,317 ± 6106	911.0	P1= <0.011*
Category II	19,726 ±6577	477.0	P2 = <0.011*
Category III	19,149 ±6384	358.0	P3 = 0.28
P value	0.89	<0.021*	

## Discussion

The carious dentin is removed by employing excavators, a high-speed handpiece device, and a low-speed handpiece device in accordance with traditional caries removal techniques. This technique undoubtedly increased the effectiveness and efficiency of cavity preparation, but it also comes with a number of unavoidable drawbacks, including the patients' perceived notion of discomfort; the need for local anesthesia; the elimination of both infected dentin as well as affected dentin, which unnecessarily weakens the tooth structure; damaging thermal impact on the tissue of the pulp; and the potential for traumatic pulp exposure [3]. Dentistry has developed in a way that maximizes the preservation of dental tissues. Polymer bursts and chemomechanical caries eradication are two noninvasive procedures that remove only the infected portion of dentin and preserve the normal dentin structure and normal tooth structure.

This approach also helps in reducing the discomfort brought on by the elimination of dentin affected by caries, making it easier to take children to dental services [8,9]. This study assessed and contrasted the clinical effectiveness and microbiologic effectiveness of these two noninvasive approaches to more conventional mechanical approaches for the removal of caries in primary molars. According to research published by Kuboki et al. (1983) [10], caries identification dye was employed to assess the clinical outcomes of caries elimination. It was believed to be more precise and to address the drawbacks of both visual approaches as well as tactile approaches. The tactile and visual approaches used by practitioners to identify caries were arbitrary and inconsistent. According to the present research, the numerical values of caries identification dye gradings in study participants in the polymer bur category were substantially higher than the values of caries detecting dye scores for the traditional group, indicating that the conventional approach is more clinically effective at removing cavities. This is consistent with research conducted by researchers like Prabhakar and Kiran in the year 2009. They discovered that whereas polymer burs tend to over-prepare cavities, round burs made of carbon steel eliminate carious lesions more effectively [11]. Additionally, the traditional method is more clinically effective at removing cavities than the Carisolv method because the numerical data regarding caries detection dye gradings for the conventional approach category was much smaller than the values for the Carisolv group [12].

This is in line with the findings of research carried out by Pandit et al. in 2007 and Kochhar et al. in 2011, who discovered that airotor was more effective than Carisolv at removing caries. Considering the caries detection dye levels, there was no vital variation observed between values obtained in the polymer bur category and values observed in the Carisolv category [12,13]. This is consistent with the findings of the research published by Soni et al. in 2015, who observed no vital variation in the clinical effectiveness of caries eradication comparing the polymer bur approach and the Carisolv approach [14]. Since the conventional approach has a tendency to an excessive elimination of tooth structure during cavity preparation due to a lack of touch feeling, its effectiveness in removing caries was at its highest. As a result, there was a gross, quick evacuation of tissue and less control over the entire procedure.

As a result, the operator did not always recognize when the actual clinical objective was met. Thus, the excavation process went on in the healthy dentin, eventually resulting in excessive preparation. As a result, due to excessive preparation, the tooth preparation produced by the conventional approach of caries removal appeared poorly stained or unstained by the caries detection dye [15]. To determine whether contaminated dentin was eliminated, caries detection chemicals and sensory and optical assessments can be used. However, none of them can reliably discriminate between dentin that has been impacted by caries and dentin that has been infected by it. In addition to the portion of dentin demineralized by bacterial biological substances, portions of healthy dentin, notably those at the dentin-enamel junction, can also be stained by caries detector dyes [16,17].

Inversely correlated with the brightness of the stained dentin portion were the frequencies of bacterial colonies present in caries. To avoid removing too much tissue, dentin that has a pale pink stain shouldn't be removed [18]. The observations in the present research showed that compared to the conventional category, the Carisolv category's mean working time for the eradication of cavities was much longer. This is in line with the findings published in research carried out by Pandit et al. in the year 2015, who discovered that the time duration for removal of caries is greater in the case of the Carisolv approach as compared with the traditional approach. Additionally, the time duration for the removal of caries is greater in the case of the Carisolv approach as compared to the polymer bur approach [12].

This is consistent with research conducted in 2015 by Divya et al., who observed similar findings [19]. Carisolv gel's prolonged operational period can be ascribed to its frequent applications. In our investigation, the cavity preparation was done with an average of 3-4 treatments. This was supplemented by several visual inspections and tactile sensations. Since the opaque gel made it difficult to examine, the cavity was washed before being examined. Carisolv's operational time was lengthened by each of these elements. However, there was no discernible difference in mean values of time of removal of caries between the study participants of the polymer bur category and those of the conventional approach, which is consistent with the findings described by research carried out by Medioni et al. [20] in 2016.

The conventional technique and the polymer bur were found to remove caries in about the same amount of time on average. The Face Image Scale was used to assess patient satisfaction with the treatment following the completion of the procedure. According to the study's findings, the median facial expression scale score in the study participants in the polymer bur category was considerably lower than in study participants belonging to the conventional mechanical approach category. This is in line with Allen et al. [9] findings from 2005 that the kids in the polymer bur category were happier than the kids in the mechanical group. Additionally, the Carisolv group's median facial expression scale scores were much lower than those of the traditional group.

This shows that the Carisolv group has more satisfied patients than the traditional approach. This is in line with the findings of research carried out by Chowdhry et al. in 2015, who came to the conclusion that patient satisfaction was better with the Carisolv approach in children as compared with that of the traditional mechanical approach. On the contrary, there was no vital variation observed between values of the facial expression scale in the Carisolv approach and the polymer burst approach. The level of patient satisfaction was greater in the Carisolv approach and the polymer burst approach than in the traditional mechanical approach. The eradication of dental cavities in a painless manner with no use of local anesthetic may be responsible for this. Additionally, many patients find the conventional group's method of administering local anesthetic irritating and unpleasant since it causes a tingling sensation and numbness [21].

In conjunction with the low-and high-speed handpieces' disagreeable sounds and vibrations, these factors could all have decreased the level of satisfaction among patients. The findings of the microbiological evaluation of this study showed that the number of living colonies of bacteria was greater in the polymer bur approach as compared to the Carisolv approach after the eradication of dental caries. This indicates that the Carisolv approach was superior to the polymer bur in terms of microbiologically reducing the number of germs present. This is consistent with the findings of research by Divya et al. in 2015, who discovered that the Carisolv approach was superior to the polymer bur approach in terms of caries removal microbiologically [19].

Additionally, our study's findings showed that the number of bacterial colonies following caries removal was much greater in the polymer bur approach than that observed in the conventional approach. It shows that the polymer bur approach was less successful than the traditional mechanical approach at removing dental caries from bacteria. This is in line with the findings of a study carried out by Hassan et al. [22], who discovered that the traditional technique was superior to the polymer bur in terms of microbiological caries eradication. However, there was no discernible difference observed between the study participants of the conventional group and the study participants of the Carisolv group in terms of the total number of living bacterial colonies following caries eradication. The findings of research carried out by Azrak et al. and Subramaniam et al. support the findings of the present research [23,24].

The study limitations are that the sample size is small and just one technique is compared with the traditional approach. The bacterial count of specific bacteria has not been compared in the study.

## Conclusions

The mechanical approach has the highest clinical efficacy for removing caries. Carisolv required the most time to remove cavities. Compared to the mechanical approach, patient satisfaction level was greater with Carisolv. It was also observed that Carisolv as well as the mechanical technique have greater antibacterial effectiveness
